# Rural-urban determinants of vitamin a deficiency among under 5 children in Bangladesh: Evidence from National Survey 2017–18

**DOI:** 10.1186/s12889-021-11607-w

**Published:** 2021-08-19

**Authors:** Md. Moyazzem Hossain, Sabina Yeasmin, Faruq Abdulla, Azizur Rahman

**Affiliations:** 1 Department of Statistics, Jahangirnagar University, Savar, Dhaka-1342, Bangladesh; 2 Department of Statistics, Faculty of Sciences, Islamic University, Kushtia-7003, Bangladesh; 3 School of Computing, Mathematics and Engineering, Charles Sturt University, Wagga Wagga, NSW 2678, Australia

**Keywords:** Vitamin a deficiency, Children, Chi-square test, Logistic regression, Bangladesh

## Abstract

**Background:**

Vitamin A supplementation reduces child morbidity, mortality, and blindness of people, especially in developing countries like Bangladesh. This study explores significant determinants of vitamin A deficiency among preschool children in rural and urban areas of Bangladesh.

**Methods:**

The data set was extracted from a nationally representative survey based on a cross-sectional study, the BDHS-2017-18. The base survey was conducted using a two-stage stratified sample of households. A sample of 8364 (urban 2911, rural 5453) children under-5 years old was analyzed using bivariate and multivariate statistical techniques.

**Results:**

Results have demonstrated that 73.9 and 73.2% of children have had a vitamin A supplementation from urban and rural areas, respectively. Logistic regression analysis showed that parents’ education plays a vital role in consuming vitamin A supplements in urban and rural areas. Children whose mothers have secondary (OR: 1.17, CI: 0.76–1.81) and higher (OR: 1.21, CI: 0.72–2.04) education were more likely to consume vitamin A supplementation than children whose mothers were illiterate in urban areas. However, in rural areas, children whose mothers have secondary education were about 24% and higher education with 60% more likely to consume vitamin A supplementation than children whose mothers were illiterate. Child’s age, regional variation and wealth index also contributing factors for vitamin A deficiency in Bangladesh.

**Conclusions:**

These findings indicated that the consumption of vitamin A does not cover the target of sustainable development goals. Thus special national and community level efforts are required to ensure the coverage of the national vitamin A program is increased adequately to the most vulnerable groups of children in Bangladesh.

## Introduction

Vitamin A is an essential nutrient needed in small amounts and mainly required for the visual system, growth and development, maintaining epithelial cellular integrity, immune function and reproduction [1]. Inadequate intake of vitamin A is the primary root of vitamin A deficiency (VAD). Nowadays, VAD is one of the most important concerns among the public health problems since many children, and pregnant women are affected by Vitamin A deficiency disorder throughout the developing countries including Bangladesh [2, 3]. Moreover, the Vitamin A deficiency (VAD) disorders reduce the immune competence, which is responsible for increasing the morbidity and mortality associated with night blindness, corneal ulcers, keratomalacia and related ocular signs and symptoms xerophthalmia [4–9]. Vitamin A deficiency is also associated with an increased risk of child mortality and of a range of problems, including measles, diarrhea, respiratory diseases, vision problems, and can lead to death [2, 10–15]. The evidence above ensures that the prevalence of many childhood diseases is more common among stunted children, and on the other hand, there is a significant association between stunting and VAD [16]. Vitamin A deficiency in under-five children can also cause mortality risk up to 20–30% [17]. Also, Xerophthalmia among preschool children amounts to about 5 million cases, 10% of which can potentially lead to blindness in India [18, 19]. Globally, about 30% of children under the age of 5 years are vitamin A deficient, and about 2% of all deaths are attributable to VAD in this age group [14, 20]. The most important agenda of Sustainable Development Goal-3 is to reduce the preventable deaths of under-five children at least as low as 25 per 1000 live births within 2030 [21] and reduce the number of stunted children under five by 40% by 2025 [22]. Therefore, vitamin A supplementation was suggested to reduce child morbidity and mortality from various diseases; however, it would be treated as a cost-effective strategy for reaching SDG-3 [23].

Since the vitamin A supplementation program is a cost-effective approach to lessen the burden of VAD, it is necessary to design the community-based vitamin A supplement program considering associated factors that significantly enhance the awareness among underprivileged people and the consumption rate of vitamin A supplementation (VAS) [24–27]. Existing literature riched by such studies that have been conducted to identify the determinants that are significantly associated with the VAS program in several countries. Abedin et al. (2019) mentioned that age, sex, religion, mother’s education, immunization status for the vaccine-preventable disease are associated with the recent receipt of VAS [21]. Mostafa et al. (2019) try to determine the factors affecting low coverage of the VAS program among the young children admitted to a diarrheal hospital of Bangladesh and age of the child, parental education, and wealth status are associated with receiving the vitamin A supplement. They also observed an increase in coverage of VAS from 61 to 76% over the last 18 years [20]. Muliyil et al. (2019) mention that the educational status of the household head was significantly associated with an increased risk of VAD among children [28]. Other studies across the world also reveal that age, sex, BMI, religion, age of mother, mother’s education, family type, occupation, income, food habits, type of residence, access to media, wealth index, and social development status of their state of residence are associated with recent receipt of VAS [2, 29–35].

However, in light of the existing literature, a substantial research gap has been discovered in this topic, particularly in Bangladesh. Therefore, to fill this gap, the authors were motivated to conduct a study to ascertain the influential determinants of VAD in urban-rural settings of Bangladesh based on the most recent BDHS-2017/18 survey data. To assess the association between the children’s vitamin A capsule consumption status and socio-economic and demographic characteristics, we used the Chi-square test in the bivariate analysis. Moreover, the logistic regression analysis was carried out to determine the effects of the covariates on the response variable.

## Methods

### Data sources, study design and participants

This study considers a secondary dataset collected from a county representative entitled Bangladesh Demographic and Health Survey (BDHS)-2017–18. The sampling frame of this survey was the list of enumeration areas (EAs) of the 2011 Population and Housing Census of the People’s Republic of Bangladesh. The primary sampling unit of this survey was an EA. The survey used two-stage stratified sampling techniques. In the first stage, 675 EAs were chosen, with 227 and 448 EAs from urban and rural areas. However, data was not possible to collect from 3 EAs due to natural disasters. These clusters were in Dhaka (one urban cluster), Rajshahi (one rural cluster), and Rangpur (one rural cluster). In the second stage of sampling, a systematic sample of 30 households selected from each EA. As a result, 20,250 residential households were selected into the four phases. Among the 20,376 ever-married women age 15–49 years eligible for interviews, 20,127 were interviewed, yielding a response rate of 99%. A sample of 8364 children aged under-5 years (5453 and 2911 from rural and urban areas, respectively) was used for the subsequent analysis. The Details of the sampling procedure are available on the report of the Bangladesh Demographic and Health Survey-2017-18 [36].

### Variables

This study aims to measure the prevalence of vitamin A capsules consumption by under-5 years’ old children in rural and urban areas of Bangladesh. The following question was asked to the child’s mother “did your child consume vitamin A capsule within the last six months?” to assess the consumption of vitamin A capsule. This question’s outcome is considered as the dependent variable, which is a dichotomous variable indicating whether a child consumed vitamin A or not. Moreover, the socio-economic and demographic characteristics like age of the child (in months), child’s sex, region/division, religion, number of children, mother’s age, mother’s education, father’s education, sex of household head, wealth index and breastfeeding status included in the subsequent analysis as a set of covariates.

### Statistical analysis

The Chi-square test is employed to determine the significance of exposure variables with the target variable, and logistic regression analysis is used to determine the influence of the selected covariates on the response variable. The results presented in odds ratios (ORs), with 95% confidence intervals (95% CIs). The IBM SPSS version 25 was utilised in all statistical analyses.

The logistic regression model [37, 38] can be expressed as,

$$ \Pr\;\left({Y}_i=1\right)=\frac{\exp\;\left({X}_i\beta \right)}{1+\exp\;\left({X}_i\beta \right)}, $$

where, *Y*_*i*_ is a binary variable that takes a value of ‘ 1 ’ if the respondent received the vitamin A capsules and ‘ 0 ’ otherwise, *X*_*i*_ is a vector of independent variables and *β* is a vector of unknown parameters which contains the intercept parameter and the regression parameters associated with a set of covariates used in the study.

The fitted form of the model can be defined as,

$$ \ln\;\left[\frac{{\hat{P}}_i}{1-{\hat{P}}_i}\right]={\hat{\beta}}_0+{\hat{\beta}}_1{X}_1+\dots +{\hat{\beta}}_k{X}_k $$

where, ${\hat{\beta}}_l\ \left(l=0,1,2,\dots, k\right)$ represents the estimated regression coefficient of the *l*^*th*^ independent variable in the study.

## Results

This section offers empirical data analysis results with descriptive interpretations. Table 1 presents the study children’s background, socioeconomic and demographic characteristics, and their distribution by the vitamin A capsule consumption status according to rural and urban areas. About 73.2 and 73.9% of children had consumed vitamin A capsules in the rural and urban areas, respectively, i.e. a less coverage of vitamin A supplementation in rural areas than in urban areas. The highest percentage (77.6%) of children consumed Vitamin A capsules in the Rangpur division and the lowest (69.6%) in the Dhaka division for rural areas. In urban areas, the highest 78.1% of children consumed Vitamin A in Mymensing and Rajshahi division and the lowest 70.2% in the Dhaka division. Thus, the division has a significant association with vitamin A consumption for Bangladesh’s urban and rural areas [Table 1].

**Table 1:** Frequency distribution of the consumption of vitamin A supplementation status according to selected characteristics in Rural and Urban areas of Bangladesh

Characteristics of the study sample	Vitamin A capsule consumption status			
	Urban children		Rural children	
	No (%)	Yes (%)	No (%)	Yes (%)
***Division*** (^a*, b**^)				
Barisal	65 (24.9)	196 (75.1)	180 (29.9)	422 (70.1)
Chittagong	114 (23.8)	366 (76.3)	228 (25.2)	678 (74.8)
Dhaka	211 (29.8)	496 (70.2)	161 (30.4)	369 (69.6)
Khulna	71 (22.3)	248 (77.7)	150 (27.4)	397 (72.6)
Mymensing	55 (21.9)	196 (78.1)	185 (25.1)	553 (74.9)
Rajshahi	76 (29.1)	185 (78.1)	153 (25.1)	456 (74.9)
Rangpur	71 (25.2)	211 (74.8)	145 (22.4)	503 (77.6)
Sylhet	97 (27.7)	253 (72.3)	259 (29.7)	614 (70.3)
***Religion*** (^a*, b**^)				
Islam	711 (26.6)	1961 (73.4)	1364 (27.3)	3624 (72.7)
Other than Islam	49 (20.5)	190 (79.5)	97 (20.9)	368 (79.1)
***Sex of household head*** (^a, b^)				
Male	689 (26.2)	1945 (73.8)	1268 (27.1)	3416 (72.9)
Female	71 (25.6)	206 (74.4)	193 (25.1)	576 (74.9)
***Wealth index*** (^a*, b^)				
Poorest	86 (28.1)	220 (71.9)	422 (27.7)	1102 (72.3)
Poor	66 (27.6)	173 (72.4)	387 (26.9)	1052 (73.1)
Middle	101 (26.0)	287 (74.0)	301 (27.3)	802 (72.7)
Richer	221 (28.4)	557 (71.6)	230 (26.2)	647 (73.8)
Richest	286 (23.8)	914 (76.2)	121 (23.7)	389 (76.3)
***Child’s sex*** (^a*, b^)				
Male	381 (26.0)	1083 (74.0)	756 (26.1)	2136 (73.9)
Female	379 (26.2)	1068 (73.8)	705 (27.5)	1856 (72.5)
***Age of Child*** (^a***, b***^)				
06–12 months	91 (30.3)	209 (69.7)	191 (29.4)	459 (70.6)
13–24 months	88 (15.5)	481 (84.5)	184 (16.6)	923 (83.4)
25–36 months	68 (12.3)	487 (87.7)	190 (18.0)	863 (82.0)
37–48 months	115 (21.5)	420 (78.5)	197 (19.0)	840 (81.0)
49–59 months	111 (19.9)	446 (80.1)	209 (21.8)	748 (78.2)
***Number of children*** (^a*, b***^)				
1 child	470 (25.2)	1395 (74.8)	756 (24.2)	2363 (75.8)
2 child	186 (26.5)	516 (73.5)	447 (28.9)	1099 (71.1)
3 or more child	34 (33.0)	69 (67.0)	94 (30.4)	215 (69.6)
***Mother’s age*** (^a***, b***^)				
15–20 years	159 (33.4)	317 (66.6)	338 (32.2)	713 (67.8)
21–25 years	253 (25.8)	728 (74.2)	523 (26.6)	1446 (73.4)
26–30 years	182 (22.8)	615 (77.2)	364 (26.2)	1027 (73.8)
31–35 years	116 (26.0)	331 (74.0)	166 (22.5)	571 (77.5)
35 or more years	50 (23.8)	160 (76.2)	70 (23.0)	235 (77.0)
***Mother’s education*** (^a***, b**^)				
No education	61 (31.4)	133 (68.6)	118 (29.1)	287 (70.9)
Primary	223 (30.7)	504 (69.3)	485 (28.8)	1198 (71.2)
Secondary	306 (24.2)	958 (75.8)	703 (26.3)	1974 (73.7)
Higher	170 (23.4)	556 (76.6)	155 (22.5)	533 (77.5)
***Father’s education*** (^a**, b*^)				
No education	103 (32.3)	216 (67.7)	250 (26.9)	678 (73.1)
Primary	225 (27.0)	608 (73.0)	544 (27.7)	1422 (72.3)
Secondary	235 (26.1)	667 (73.9)	473 (27.4)	1253 (72.6)
Higher	182 (22.8)	618 (77.3)	169 (22.6)	578 (77.4)
***Breastfeeding status*** (^a***, b***^)				
No	247 (18.7)	1075 (81.3)	409 (19.7)	1672 (80.3)
Yes	513 (32.3)	1076 (67.7)	1052 (31.2)	2320 (68.8)

^a^represent the significance level associated with rural area children;

^b^represent the significance level associated with urban area children;

*p < 0.10; **p < 0.05; and ***p < 0.01

The age of the child and religious status factors are significantly related to the consumption status of Vitamin A capsules in both urban and rural areas. Looking at the age group of 6–12 months, 70.6% of children in rural areas have consumed vitamin A capsules whereas, in the urban area, only 69.7% of children had consumed vitamin A capsules. This result indicates that the child lives in urban and rural areas are same likely to consume vitamin A supplement, however, in the previous survey, the child who lived in rural areas were more vulnerable to vitamin A deficiency-related diseases than child lived in urban areas. Parental education plays a vital role in vitamin A consumption by their child. Both in rural and urban areas, vitamin A consumption increased with the increase of wealth index. Breastfeeding children were less consuming vitamin A capsules than their counterparts [Table 1].

**Fig. 1:**
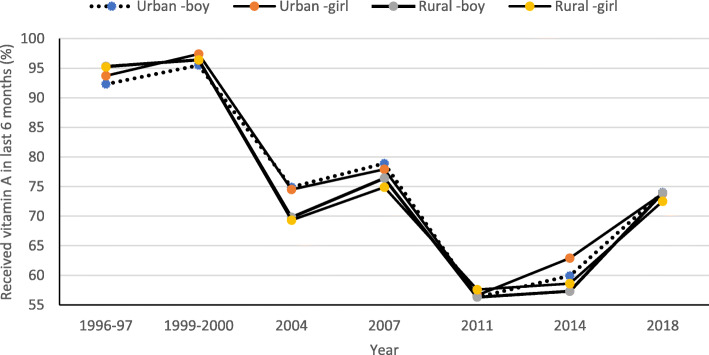
Rural-urban trends of consuming vitamin A capsule within the last six months by Sex of under-five children in Bangladesh, 1996–2018

The prevalence of vitamin A consumption was more than 90% in the 1996–97 survey. It increased around 95% in the 1999–2000 survey for boys and girls in urban and rural areas of Bangladesh. After the year 2000, the rate of receiving the vitamin A supplement by children decreased dramatically for all groups. In 2004, the prevalence of vitamin A consumption of urban boys and girls was slightly higher than rural boys and girls. The lowest figures observed in 2011 for all categories, and then the estimates increased a bit in the 2014 survey, especially for the estimates of both boys and girls in urban areas. The vitamin A coverage again increased in 2018 compared to the previous survey year 2014, both in urban and rural areas among both sexes. In general, findings demonstrated that girls received more vitamin A capsules than boys, and the prevalence of receiving vitamin A was slightly more in urban areas than in rural areas of Bangladesh. An overall downward trend of the prevalence of vitamin A consumption is observed with some fluctuations for 1996 to 2018 by the child’s sex and rural-urban status (see Fig. 1).

**Fig. 2:**
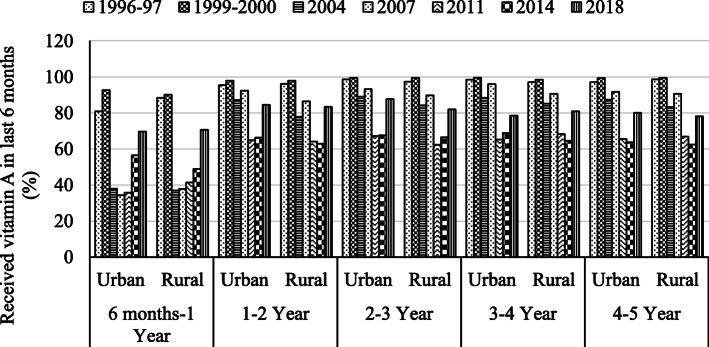
Rural-urban trends of consuming vitamin A capsule within the last six months by the age of under-five children in Bangladesh, 1996–2018

The bar graph presented in Fig. 2 depicts the comparison of receiving the vitamin A supplement in the last 6 months by the urban-rural setting of different age groups of children from 1996 to 2018. In the age category of 6 months to 1 year, the prevalence was almost double in 1996–96 and 1999–2000 than the remaining years. A slight variation is observed by region as well as the age of the child. Surprisingly the overall rate of consumption of vitamin A supplements has been decreased in recent years for children aged between one and 5 years (see, Fig. 2). Also, for the age group of 6 months to 1 year, this study observed the lowest percentage of the child received vitamin A capsules in the last one and half decades in urban and rural areas of Bangladesh.

In urban areas of Bangladesh, children aged 13–24 months (OR: 2.33; CI: 1.56–3.42) and 25–36 months (OR: 0.82; CI: 0.57–1.19) were more and less likely to consume vitamin A in comparison to the age group 6–12 months respectively. The results revealed that with the increasing age of a child, consuming vitamin A supplements decreases in both urban and rural areas. Children whose mothers have secondary (OR: 1.17, CI: 0.76–1.81) and higher (OR: 1.21, CI: 0.72–2.04) education were more likely to consume vitamin A supplementation than children whose mothers were illiterate in urban areas (see in Table 2). However, in rural areas, children whose mothers have secondary education were about 24% (OR: 1.24, CI: 0.92–1.68) and higher education with 60% (OR: 1.60, CI: 1.07–2.38) more likely to consume vitamin A supplementation than children whose mothers were illiterate. Somewhat similar results were observed for the father’s education. These results depict that parents education play a vital role in the consumption of vitamin A supplement. Rural children living in different divisions received vitamin A capsules more than the Barisal division except for Dhaka and Sylhet divisions. Moreover, children with middle and upper-class families in urban and rural areas were more likely to receive vitamin A supplementation than children with low-income families. However, wealthier people are less concerned about vitamin A consumption by their children. In contrast, for the urban areas, children aged 13–24 months and 24–36 months were more likely to consume vitamin A than the age group 6–12 months [Table 2].

**Table 2:** Logistic regression estimates and odds ratio of different socioeconomic and demographic variables on vitamin A deficiency in Rural and Urban areas of Bangladesh, 2017–18

Characteristic	Vitamin A deficiency in Urban area			Vitamin A deficiency in Rural area		
	Coefficient (β)	SE (β)	Odds ratio of β (95% CI)	Coefficient (β)	SE (β)	Odds ratio of β (95% CI)
**Division**						
Barisal (ref)	–	–	–	–	–	–
Chittagong	−0.06	0.22	0.94 (0.61, 1.45)	0.23	0.14	1.26 (0.95, 1.66)
Dhaka	−0.38*	0.21	0.68 (0.45, 1.03)	−0.12	0.16	0.89 (0.66, 1.2)
Khulna	0.03	0.24	1.03 (0.64, 1.64)	0.13	0.16	1.14 (0.84, 1.55)
Mymensing	0.10	0.25	1.1 (0.68, 1.8)	0.32*	0.15	1.38 (1.03, 1.83)
Rajshahi	−0.35*	0.24	0.7 (0.44, 1.12)	0.16	0.15	1.18 (0.87, 1.59)
Rangpur	0.03	0.24	1.03 (0.64, 1.65)	0.35**	0.16	1.42 (1.05, 1.92)
Sylhet	−0.20	0.23	0.82 (0.52, 1.28)	−0.02	0.14	0.98 (0.74, 1.3)
**Religion**						
Islam (ref)	–	–	–	–	–	–
Others	0.40**	0.20	1.49 (1, 2.21)	0.33	0.14	1.39 (1.05, 1.84)
**Sex of household head**						
Male (ref)	–	–	–	–	–	–
Female	0.02	0.18	1.02 (0.71, 1.46)	0.02	0.11	1.03 (0.83, 1.27)
**Wealth index**						
Poorest (ref)	–	–	–	–	–	–
Poor	0.02*	0.24	1.02 (0.64, 1.63)	0.01*	0.10	1.01 (0.83, 1.22)
Middle	0.16*	0.22	1.17 (0.77, 1.8)	0.04*	0.11	1.04 (0.83, 1.3)
Richer	−0.20*	0.19	0.82 (0.56, 1.19)	−0.05	0.13	0.95 (0.74, 1.22)
Richest	0.06	0.21	1.06 (0.7, 1.59)	0.04	0.16	1.04 (0.76, 1.44)
**Child’s sex**						
Male (ref)	–	–	–	–	–	–
Female	0.01***	0.10	1.01 (0.83, 1.23)	−0.11*	0.07	0.9 (0.78, 1.03)
**Age of Child**						
06–12 months (ref)	–	–	–	–	–	–
13–24 months	0.84***	0.20	2.33 (1.56, 3.42)	1.12***	0.12	3.02 (1.44, 3.83)
25–36 months	−0.21**	0.19	0.82 (0.57, 1.19)	0.06*	0.11	1.06 (0.86, 1.31)
37–48 months	−0.26**	0.17	0.77 (0.55, 1.08)	−0.11**	0.10	0.91 (0.74, 1.11)
49–59 months	−0.25***	0.16	0.77 (0.56, 1.06)	−0.14**	0.09	0.87 (0.72, 1.04)
**Number of children**						
1 child (ref)	–	–	–	–	–	–
2 child	0.02	0.12	1.02 (0.8, 1.3)	−0.05	0.09	0.95 (0.8, 1.12)
3 or more child	−0.24*	0.26	0.78 (0.47, 1.31)	−0.01*	0.16	0.99 (0.73, 1.35)
**Mother’s age**						
15–20 years (ref)	–	–	–	–	–	–
21–25 years	−0.06	0.16	0.94 (0.68, 1.29)	0.01	0.11	1.01 (0.82, 1.24)
26–30 years	0.08*	0.17	1.05 (0.72, 1.39)	0.06	0.11	1.06 (0.85, 1.33)
31–35 years	−0.14*	0.19	0.87 (0.6, 1.25)	0.19*	0.13	1.21 (0.93, 1.57)
35 or more years	0.03	0.24	1.03 (0.65, 1.63)	0.19	0.18	1.2 (0.84, 1.72)
**Mother’s education**						
No education (ref)	–	–	–	–	–	–
Primary	−0.07	0.22	0.93 (0.61, 1.42)	0.04	0.15	1.04 (0.78, 1.38)
Secondary	0.16*	0.22	1.17 (0.76, 1.81)	0.22*	0.15	1.24 (0.92, 1.68)
Higher	0.19*	0.27	1.21 (0.72, 2.04)	0.47**	0.20	1.60 (1.07, 2.38)
**Father’s education**						
No education (ref)	–	–	–	–	–	–
Primary	0.14	0.17	1.15 (0.82, 1.62)	0.03	0.11	1.03 (0.84, 1.27)
Secondary	0.17*	0.19	1.19 (0.83, 1.72)	0.03	0.12	1.03 (0.82, 1.31)
Higher	0.22	0.23	1.24 (0.79, 1.94)	0.34**	0.17	1.41 (1.01, 1.96)
**Breastfeeding status**						
No (ref)	–	–	–	–	–	–
Yes	−0.19*	0.16	0.83 (0.6, 1.13)	0.03*	0.11	1.03 (0.84, 1.28)

*p < 0.10; **p < 0.05; and ***p < 0.01

## Discussion

This study demonstrated the significance of potential socioeconomic and demographic factors associated with vitamin A supplementation among under-5 children in the settings of rural and urban areas of Bangladesh. It is found that the children residing in different divisions (administrative regions) have a varying percentage of receiving vitamin A capsules- leading the division as a significant factor for receiving vitamin A capsules. This finding is also supported by another study [21]. The authors think that this variation is due to the demographic and socio-economic diversity among divisions. In this study, urban children from middle-class families are more likely to receive vitamin A supplementation than others because Bangladeshi middle-class families are more concerned about their children. Subsequently, children from the wealthiest family have a slightly higher chance of vitamin A consumption in rural and urban areas. This finding is consistent with the result of a previous study [21, 30].

Children’s age is another contributing factor, and this study revealed that the children of age group 13–24 months are higher likely to receive vitamin A capsules. However, after 24 months, children are less likely to consume vitamin A capsules than 6–12 months aged children in urban and rural areas. These results are probably due to the assorted carrying attitudes of parents or caregivers to their offspring in different age groups. However, Janmohamed et al. (2017) and Agrawal and Agrawal (2013) found that all age groups are equally likely to consume vitamin A capsules [30, 39].

This study demonstrated that parental education was key determinants for receiving vitamin A capsule in Bangladesh’s rural and urban areas, which was a consistent finding with other [2, 29]. This study found that in rural areas, children whose mother and father having higher educational qualifications (i.e., secondary and higher) are more likely to consume vitamin A capsules. However, it observed a different result in urban areas, i.e. children whose father and mother had the primary education were more likely to consume vitamin A capsules. It could be because a higher educated parent in an urban area remains busy with other activities except for their parenting task. Therefore, parents’ awareness of their children may be more effective in reducing the VAD burden [20, 25]. The educated parents’ having enough knowledge about vitamin A-rich food also plays a crucial factor in preventing the vitamin A deficiency of their offspring. A study found a significant positive association between maternal education VAS attendance in Cambodia [40]. The families having three or more children were more likely to consume vitamin A supplementation in rural and urban areas of Bangladesh. It is because elder offspring sometimes look after their younger siblings among families having more children. The authors believe that the quality of the data ensures the acceptance of the findings.

## Conclusions

This study depicted that vitamin A supplementation coverage for the urban and rural areas is 73.9 and 73.2%, respectively, which indicates that to achieve the SDGs target, we have to prioritise and improve the vitamin A campaign in Bangladesh. Findings also suggest that vitamin A supplementation is less in rural areas than the urban areas. Moreover, the results are varied significantly with region, religion, wealth index, children’s age, mother’s age, mother’s education, and father’s education in the rural area. This study examined the nature and determined the essential determinants of children’s vitamin A supplementation intake. The significant factors identified in this study will be helpful for the researcher to identify other related factors to reduce child mortality due to vitamin A deficiency.

Since the human body cannot produce vitamin A, it must be included in our diet. Therefore, the authors strongly recommended consuming a regular intake of vitamin A-rich foods such as liver, chicken, beef, eggs, oily fish, milk, mangoes, orange fruits, sweet potatoes, carrots, spinach, and other green vegetables. It is also suggested that mothers should breastfeed their child as it contains vitamin A which is essential for young children. Moreover, various fortified foods with vitamin A, like cereals, slices of bread, pastries, and crackers, may be used to supplement vitamin A. An improvement of dietary intake of vitamin-A may enhance the vitamin A status among under-5 children. Thus, dietary intake, including the foods mentioned earlier, will be considered an alternative approach to taking vitamin A supplements to prevent vitamin A deficiency.

Moreover, these findings would assist decision-makers to design effective policies that could improve some of the risk determinants, especially in the rural area, to increase concern for vitamin A supplementation. There may be a spatial variation in the data at local area levels, which could help developing precision policy and the most effective implementation [38]. The future study should focus on expanding the analysis to include other explanatory variables such as geospatial attributes related to child health and using the up-to-date data. Future research can also employ a multi-level modeling approach for minimizing any higher-order variations in the data [37, 41]. Finally, it may better understand the dynamics of vitamin A coverage in other Southeast Asian countries.

## Data Availability

After registration, the data set is available via the following access link http://dhsprogram.com/data/available-datasets.cfm.

## References

[1] Ahmed F (1999). Vitamin A deficiency in Bangladesh: A review and recommendations for improvement. Public Health Nutr.

[2] Rahman A, Sapkota M (2014). Knowledge on vitamin a rich foods among mothers of preschool children in Nepal: impacts on public health and policy concerns. Sci J Public Heal.

[3] Semba RD, de Pee S, Sun K, Akhter N, Bloem MW, Raju VK (2010). Coverage of vitamin a capsule programme in Bangladesh and risk factors associated with non-receipt of vitamin a. J Health Popul Nutr.

[4] Dixit DT (1966). Night-blindness in third trimester of pregnancy. Indian J Med Res.

[5] Rahmathullah L, Underwood BA, Thulasiraj RD, Milton RC, Ramaswamy K, Rahmathullah R, Babu G (1990). Reduced mortality among children in southern India receiving a small weekly dose of vitamin a. N Engl J Med.

[6] Sommer A, West KPJ (1996). Vitamin a deficiency: health, survival, and vision.

[7] Sommer A, Hussaini G, Tarwotjo I, Susanto D (1983). Increased mortality in children with mild vitamin a deficiency. Lancet..

[8] Sommer A, Djunaedi E, Loeden AA, Tarwotjo I, West KP, Tilden R (1986). Impact of vitamin a supplementation on childhood mortality. A randomized controlled community trial. Lancet..

[9] West KP, Katz J, LeClerq SC, Pradhan EK, Tielsch JM, Sommer A (1991). Efficacy of vitamin a in reducing preschool child mortality in Nepal. Lancet..

[10] Kassu A, Andualem B, Van Nhien N, Nakamori M, Nishikawa T, Yamamoto S (2007). Vitamin a deficiency in patients with diarrhea and HIV infection in Ethiopia. Asia Pac J Clin Nutr.

[11] Imdad A, Mayo-Wilson E, Herzer K, Bhutta ZA (2017). Vitamin a supplementation for preventing morbidity and mortality in children from six months to five years of age. Cochrane Database Syst Rev.

[12] Akhtar S, Ahmed A, Randhawa MA, Atukorala S, Arlappa N, Ismail T (2013). Prevalence of vitamin a deficiency in South Asia: causes, outcomes, and possible remedies. J Health Popul Nutr.

[13] World Health Organization (WHO) (2014). Xerophthalmia and night blindness for the assessment of clinical vitamin A deficiency in individuals and populations.

[14] Stevens GA, Bennett JE, Hennocq Q, Lu Y, De-Regil LM, Rogers L (2015). Trends and mortality effects of vitamin a deficiency in children in 138 low-income and middle-income countries between 1991 and 2013: a pooled analysis of population-based surveys. Lancet Glob Health.

[15] Wirth JP, Petry N, Tanumihardjo SA, Rogers LM, McLean E, Greig A, Garrett G, Klemm R, Rohner F (2017). Vitamin a supplementation programs and country-level evidence of vitamin a deficiency. Nutrients..

[16] Ssentongo P, Ba DM, Ssentongo AE, Fronterre C, Whalen A, Yang Y, Ericson JE, Chinchilli VM (2020). Association of vitamin a deficiency with early childhood stunting in Uganda: a populationbased cross-sectional study. PLoS One.

[17] World Health Organization (WHO). Guideline: Vitamin A supplementation in infants and children 6–59 months of age. 2011. 24575452

[18] Dole K, Gilbert C, Deshpande M, Khandekar R (2009). Prevalence and determinants of xerophthalmia in preschool children in urban slums, Pune, India - a preliminary assessment. Ophthalmic Epidemiol.

[19] Semba RD, de Pee S, Sun K, Campbell AA, Bloem MW, Raju VK (2010). Low intake of vitamin A-rich foods among children, aged 12-35 months, in India: association with malnutrition, anemia, and missed child survival interventions. Nutrition..

[20] Mostafa I, Islam SF, Mondal P, Faruque ASG, Ahmed T, Hossain MI (2019). Factors affecting low coverage of the vitamin a supplementation program among young children admitted in an urban diarrheal treatment facility in Bangladesh. Glob Health Action.

[21] Abedin MM, Maniruzzaman M, Ali M, Ahmed NF, Ahammed B (2019). Assessing and Determining Potential Factors Associated with Vitamin A Supplementation in Bangladesh. Biostat Biometrics Open Access J.

[22] Shekar M, Kakietek J, D’Alimonte MR, Rogers HE, Eberwein JD, Akuoku JK, Pereira A, Soe-Lin S, Hecht R (2017). Reaching the global target to reduce stunting: an investment framework. Health Policy Plan.

[23] Vijayaraghavan K (2018). National control programme against nutritional blindness due to vitamin a deficiency: current status & future strategy. Indian J Med Res.

[24] Mahajan H, Srivastav S, Mukherjee S (2016). Coverage of vitamin a supplementation among under-five children in an urban resettlement colony of district Gautam-Budh Nagar, Uttar Pradesh. Int J Med Sci Public Heal Online.

[25] Adamu M, Muhammad N (2016). Assessment of vitamin a supplementation coverage and associated barriers in Sokoto state, Nigeria. Ann Niger Med.

[26] Changezi F, Lindberg L (2017). Socio-economic determinants of vitamin a intake in children under 5 years of age: evidence from Pakistan. J Hum Nutr Diet.

[27] Lima RBM, Ferreira HS, Cavalcante AL, Santos LGML, Vieira RCS, Assunção ML (2020). Coverage and educational actions related to the national vitamin A supplementation program: a study in children from the state of Alagoas. J Pediatr.

[28] Muliyil DE, Rose A, Senthamizh SV, Chatterjee T, Helan J, Kang G, Muliyil J (2019). Prevalence and risk factors of vitamin a deficiency in children and women of childbearing age in a southern Indian tribal population: a cross-sectional study. Indian J Community Med.

[29] Susan C, Shashidhara YN, Kurian N (2014). Awareness of Vitamin A Supplementation Among Mothers of Under-five Children in Selected Urban and Rural Areas. Nursing (Lond).

[30] Agrawal S, Agrawal P (2013). Vitamin a supplementation among children in India: does their socioeconomic status and the economic and social development status of their state of residence make a difference?. Int J Med Public Heal.

[31] Tariku A, Fekadu A, Ferede AT, Mekonnen Abebe S, Adane AA (2016). Vitamin-a deficiency and its determinants among preschool children: a community based cross-sectional study in Ethiopia. BMC Res Notes.

[32] Huda MN, Ahmad SM, Kalanetra KM, Taft DH, Alam MJ, Khanam A, Raqib R, Underwood MA, Mills DA, Stephensen CB (2019). Neonatal vitamin a supplementation and vitamin a status are associated with gut microbiome composition in Bangladeshi infants in early infancy and at 2 years of age. J Nutr.

[33] Roy R, Gupta A, Chaudhry M (2016). Prevalence of vitamin a deficiency in school children aged 6-16 years in Taoru tehsil of South Haryana. Int J Sci Reports.

[34] Rahman S, Rahman AS, Alam N, Ahmed AS, Ireen S, Chowdhury IA (2017). Vitamin a deficiency and determinants of vitamin a status in Bangladeshi children and women: findings of a national survey. Public Health Nutr.

[35] Marjan N, Rahman A, Rois R, Rahman A (2021). Factors associated with coverage of vitamin a supplementation among Bangladeshi children: mixed modelling approach. BMC Public Health.

[36] National Institute of Population Research and Training (NIPORT), ICF (2020). Bangladesh Demographic and Health Survey 2017–18.

[37] Rahman A (2017). Estimating small area health-related characteristics of populations: a methodological review. Geospat Health.

[38] Rahman A, Harding A (2017). Small Area Estimation and Microsimulation Modeling.

[39] Janmohamed A, Klemm RDW, Doledec D (2017). Determinants of successful vitamin a supplementation coverage among children aged 6-59 months in thirteen sub-Saharan African countries. Public Health Nutr.

[40] Grover DS, De Pee S, Ms KS, Raju VK, Bloem MW, Semba RD (2008). Vitamin a supplementation in Cambodia: program coverage and association with greater maternal formal education. Asia Pac J Clin Nutr.

[41] Das S, Rahman A, Ahamed A, Rahman ST (2019). Multi-level models can benefit from minimizing higher-order variations: an illustration using child malnutrition data. J Stat Comput Simul.

